# Supplementary material for: Improved genome assembly of double haploid Prunus persica siblings ‘Lovell 2D’ and ‘Lovell 5D’ and the peach NLRome
Source: Sci Rep. 2026 Apr 21;16:18553. doi: 10.1038/s41598-026-46952-6 (PMC13269891; doi:10.1038/s41598-026-46952-6)
Supplement: Supplementary file 1 — Supplementary Material 1 [file 41598_2026_46952_MOESM1_ESM.docx]

**Supplementary Figure 1.** Nearest NLR neighbors in A) Lovell_5D, B) Prup, C) Ppcc, D) CN14, E) 124Pan, with the distance to the nearest NBARC shown in red, the distance to the nearest NBARC for singletons (those NBARCs unique to Lovell 2D v3) in green, the distance of NBARCs to the nearest coding gene in blue, and the distance of singleton NBARCs to the nearest coding gene in purple.

**
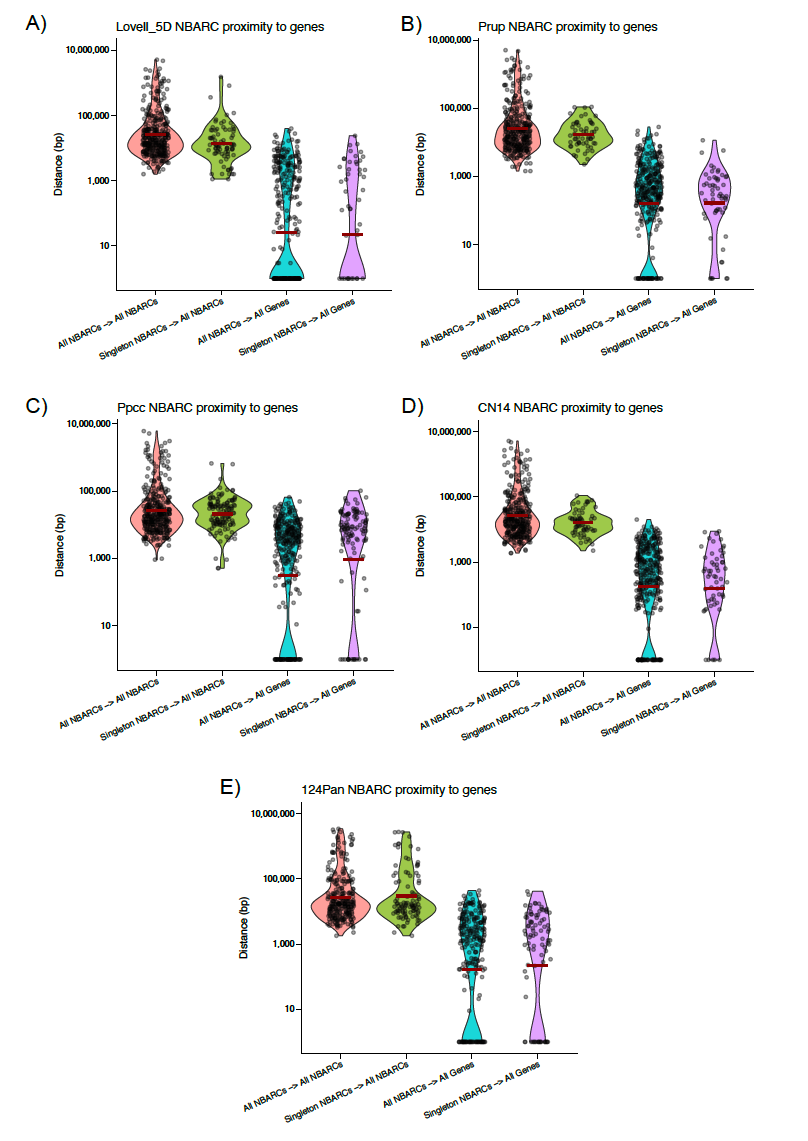
**

**Supplementary Figure 2.** Structural variation and synteny between the reference ‘Lovell 2D’ genome and ‘Lovell 5D’ HiFi-only assembly.


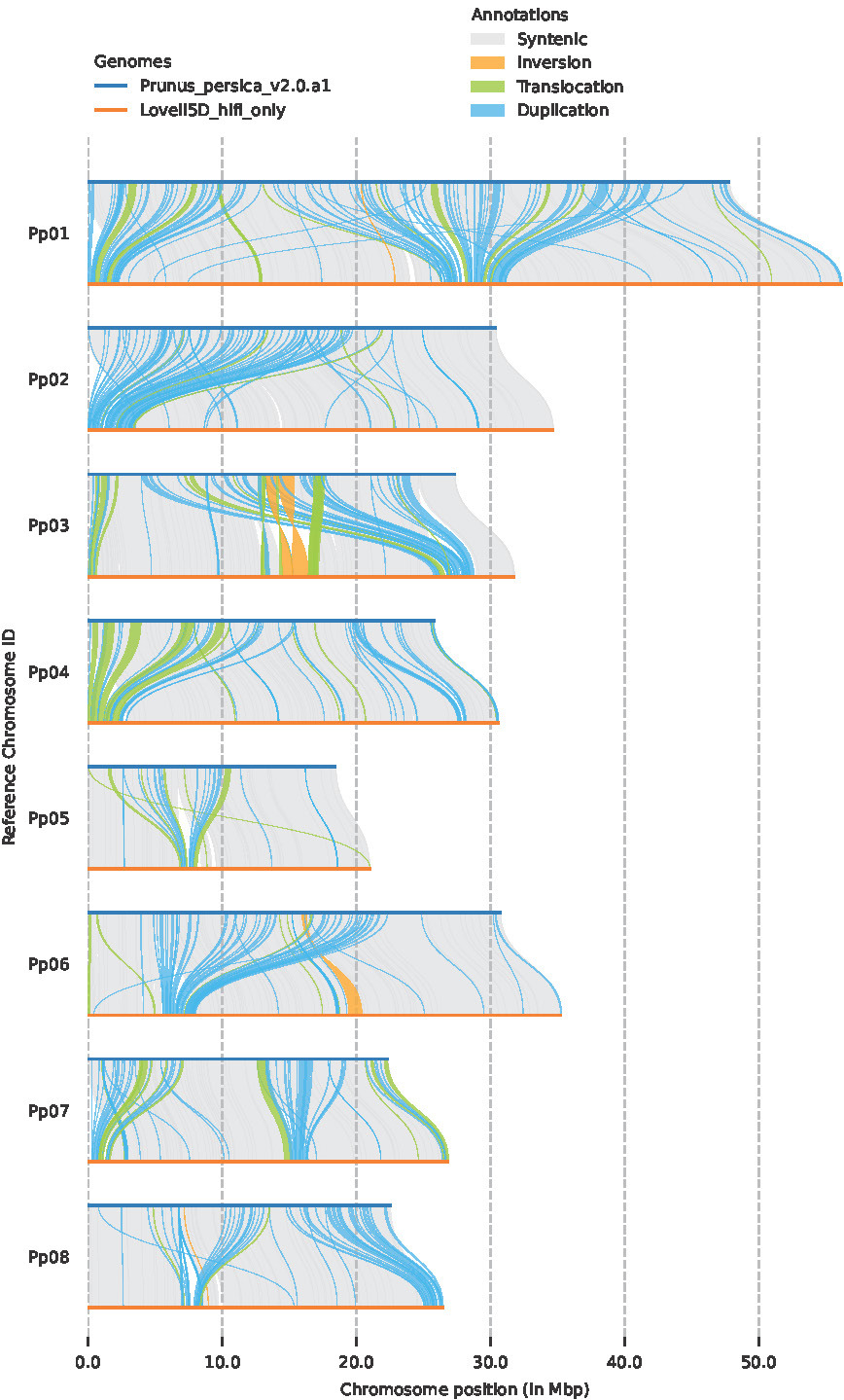


**Supplementary Figure 3.** Genome browser screenshots of ‘Lovell 5D’ HiFi read alignments on the ‘Lovell 2D’ v3.0 genome assembly of the first 1.2 Mb of Pp04 (Top) and Pp06 (Bottom).


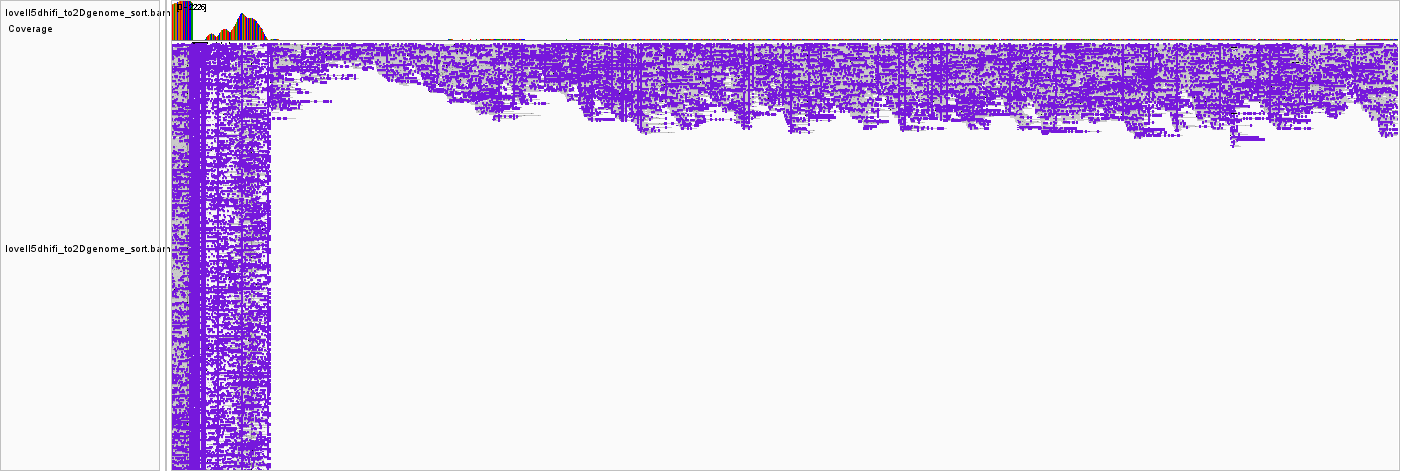

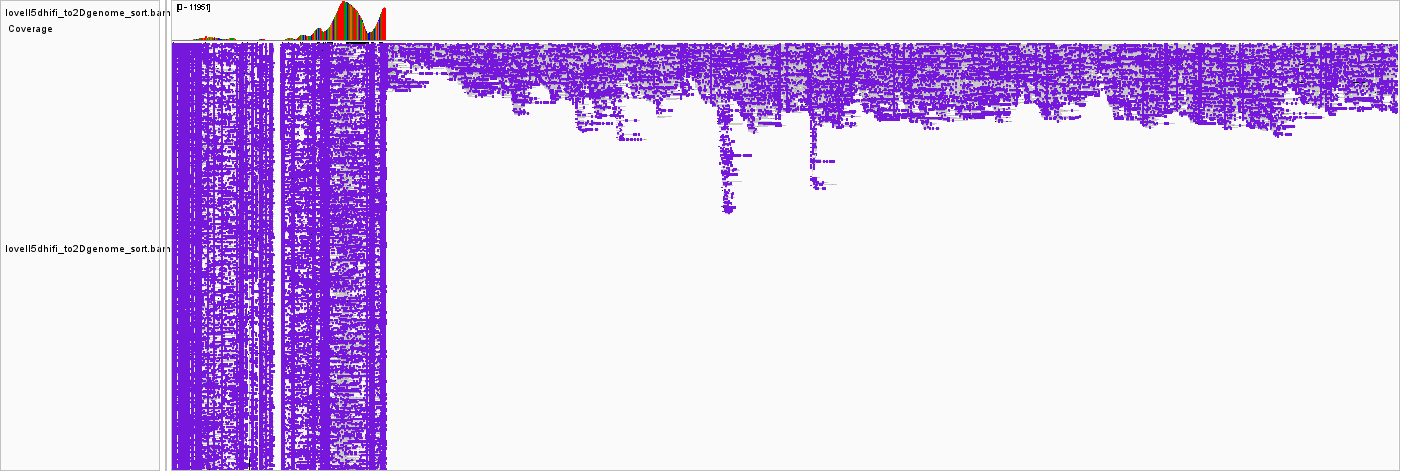


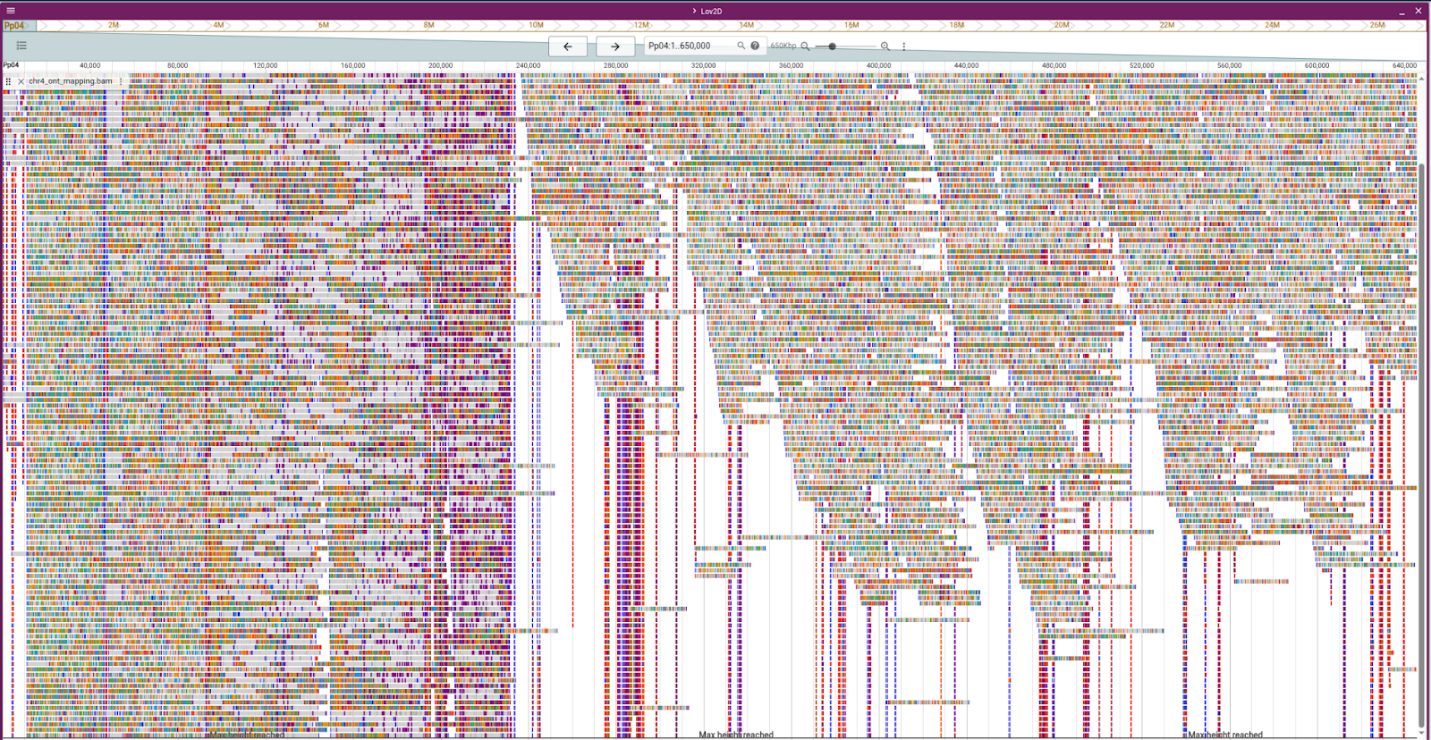


**Supplementary Figure 4.** Genome browser screenshots of ‘Lovell 5D’ ONT read alignments on the ‘Lovell 2D’ v3.0 genome assembly of the first 1.2 Mb of Pp04.
